# Real-world evaluation of persistence, effectiveness and usage patterns of tofacitinib in treatment of psoriatic arthritis in Australia

**DOI:** 10.1007/s10067-024-06930-7

**Published:** 2024-03-09

**Authors:** Geoffrey Littlejohn, Joanna Leadbetter, Belinda E. Butcher, Marie Feletar, Catherine O’Sullivan, Tegan Smith, David Witcombe, Ho Yin Ng, Peter Youssef

**Affiliations:** 1 OPAL Rheumatology Ltd, Sydney, NSW Australia; 2 Departments of Medicine, Monash Medical Centre, Monash University and Rheumatology, Monash Health, Clayton, VIC 3168 Australia; 3 WriteSource Medical Pty Ltd, Lane Cove, New South Wales, Australia; 4 School of Biomedical Sciences, University of New South Wales, Kensington, NSW Australia; 5 Rheumatology, Dandenong, VIC Australia; 6 Pfizer Australia, Sydney, NSW Australia; 7 Institute of Musculoskeletal Health at University of Sydney, Sydney, NSW Australia; 8 Royal Prince Alfred Hospital, Camperdown, NSW Australia

**Keywords:** bDMARDs, Psoriatic arthritis, Real-world, Tofacitinib, Treatment persistence

## Abstract

**Objectives:**

To describe treatment patterns and persistence of tofacitinib, interleukin 17 inhibitors (IL-17Ai) and tumour necrosis factor inhibitors (TNFi), in patients with psoriatic arthritis (PsA).

**Methods:**

Data from adult patients with PsA and who had received at least one prescription of tofacitinib, IL-17Ai or TNFi between May 2019 and September 2021 were sourced from the Australian OPAL dataset. Persistence, analysed via Kaplan–Meier methods, and propensity score matching between tofacitinib and bDMARD (IL-17Ai and TNFi) groups were conducted.

**Results:**

Of 16,692 patients with PsA, 1486 (*n* = 406 tofacitinib, *n* = 416 IL-17Ai and *n* = 664 TNFi) were included. More females were in the tofacitinib group (75.4%) than in the IL-17Ai (61.1%) and TNFi (64.8%) groups. Overall, 19.2% of tofacitinib patients were first line, compared with 41.8% of IL-17Ai and 62.8% of TNFi patients. In the overall population, the median persistence was 16.5 months (95% CI 13.8 to 19.5 months), 17.7 months (95% CI 15.8 to 19.6 months) and 17.2 months (95% CI 14.9 to 20.5 months) in the tofacitinib, IL-17Ai and TNFi groups, respectively. Persistence was similar in the tofacitinib/IL-17Ai matched population; however, in the tofacitinib/TNFi matched population, persistence was longer in the tofacitinib group (18.7 months, 95% CI 15.6 to 21.4 months) compared with the TNFi group (12.2 months, 95% CI 19.9 to 14.9 months).

**Conclusions:**

In this Australian real-world dataset, tofacitinib was more frequently used in later lines and among a slightly higher proportion of female patients than IL-17Ai or TNFi. Overall, treatment persistence was similar for tofacitinib, IL-17Ai and TNFi, but tofacitinib exhibited longer persistence than TNFi in a matched population.

**Table:** 

**Key Points** • *This is the first, large real-world study from Australia investigating the demographics, treatment patterns and comparative treatment persistence of patients with psoriatic arthritis (PsA) treated with tofacitinib and biologic disease-modifying drugs (bDMARDs).* • *The study suggests that tofacitinib is an effective intervention in PsA with at least comparable persistence to bDMARDs: tumour necrosis factor inhibitors (TNFi) and interleukin-17 A inhibitors (IL-17Ai).*

**Supplementary Information:**

The online version contains supplementary material available at 10.1007/s10067-024-06930-7.

## Introduction

Psoriatic arthritis (PsA) is an immune-mediated inflammatory condition associated with musculoskeletal manifestations and psoriasis (PsO). Global estimates of the prevalence of PsA vary from 0.3 to 1% of the population [1] and are typically reported to affect males and females equally between the ages of 40 and 50 years [2]. PsA is clinically heterogeneous with patients often presenting with a variety of symptoms including peripheral arthritis, axial disease, enthesitis, dactylitis, PsO, nail disease (pitting, onycholysis, hyperkeratosis, etc.), uveitis and inflammatory bowel disease (IBD) [3, 4]. Common comorbidities such as osteoporosis, diabetes and cardiovascular disease are also associated with increased mortality risk in patients with PsA [5, 6]. Although not fully elucidated, aberrant activation of the immune system and hyper-expression of pro-inflammatory cytokines are believed to play a key role in the pathogenesis of PsA [7].

The diversity of musculoskeletal manifestations and the presence of comorbidities can make the management of PsA complex. Fortunately, advances in therapies, namely the biological disease-modifying anti-rheumatic drugs (bDMARDs) and more recently, the newer class of targeted synthetic DMARDS (tsDMARDs), the Janus kinase inhibitors (JAKi), have provided potent tools with which to control many facets of the disease. The bDMARD therapies available for the treatment of PsA act to block some of the major pro-inflammatory cytokines implicated in the pathogenesis of this disease, namely tumour necrosis factor α (TNF α), interleukin 17A (IL-17A), interleukin 12/23 (IL-12/23) and interleukin 23 (IL-23) [8]. Janus kinases are important intracellular tyrosine kinases (JAK1, JAK2, JAK3 and Tyk2 (tyrosine kinase 2)) that also directly or indirectly regulate many of the cytokines involved in PsA. Tofacitinib was the first JAKi approved and reimbursed in Australia for the treatment of PsA in 2019 and in cellular settings preferentially inhibits JAK1 and JAK3, with lesser activity at JAK2 [9, 10]. Since then, upadacitinib has been approved for reimbursement but was not available for the treatment of PsA during the timeframe of this study.

While the efficacy and safety of tofacitinib at improving the signs and symptoms of active PsA were shown in the two global phase 3 studies; Oral Psoriatic Arthritis Trial (OPAL) Broaden and OPAL Beyond [11, 12], real-world data describing the characteristics and outcomes of patients who receive tofacitinib for the management of PsA is limited.

In Australia, the cost of b/tsDMARD therapy for the treatment of PsA is subsided by the Pharmaceutical Benefits Scheme (PBS) if a patient has severe PsA with demonstratable active disease.

OPAL Rheumatology is a consortium of Australian rheumatologists who use a bespoke electronic medical record (EMR) in their routine clinical practice. This study aimed to use the OPAL dataset to provide real-world evidence on the clinical effectiveness, treatment persistence and treatment patterns, for patients with PsA being treated with tofacitinib in the post-approval setting. Similar data were collected for patients treated with bDMARDs to provide context in a large real-world clinical practice setting.

## Methods

### Study design and setting

This was a retrospective cohort study describing the treatment persistence, clinical effectiveness and treatment patterns in patients with PsA prescribed tofacitinib or bDMARDs. At present 112 rheumatologists (approximately one-third of Australian rheumatologists) practising in 43 predominantly private clinics around Australia are contributing their clinical records to this initiative. De-identified clinical data captured at the point-of-care is extracted from all participating sites on a quarterly basis and aggregated to create the Optimising Patient outcomes in Australian rheumatoLogy (OPAL) dataset [13]. Clinical information captured during routine consultations was entered into patients’ EMR (Audit4, Software4Specialists Pty Ltd, Australia) by the clinician. Pathology reports were electronically transferred from pathology providers and deposited into the record. Information on the dataset has been published previously [13]. Diagnoses were made by individual rheumatologists rather than being criteria based. Data de-identified for patient, clinic and clinician were exported from each OPAL member’s local server, aggregated across all sites and analysed based on a predefined protocol.

The activities of OPAL Rheumatology Ltd have received overarching ethics approval from the University of New South Wales (UNSW) Human Research Ethics Committee (HREC), based on a patient opt-out arrangement (HC17799). This research protocol was approved by the UNSW HREC (HC200786).

### Participants

Patients were included if they were registered in the OPAL dataset with a clinical diagnosis of PsA, were aged between 18 and 95 years of age and had received a prescription for a bDMARD or tsDMARD in the period 01 May 2019 to 30 September 2020. There was a minimum of 1-year follow-up for all sampled patients and therefore data up to 30 September 2021 were included in the study. Minimum follow-up in each group was 12 months (by design) and the median was 22 months in each group. The b/tsDMARDs that were approved for use in Australia and included in this study were adalimumab, certolizumab pegol, etanercept, golimumab, infliximab, ixekizumab, secukinumab, tofacitinib and ustekinumab. Tofacitinib was the only tsDMARD included as no other JAKi were approved in Australia at the time of the study. Although patients on b/tsDMARDs other than tofacitinib, IL-17Ai or TNFi were planned to be included in the analyses there were only a few patients on ustekinumab, and according to OPALs Data Governance Policy, results from these patients were not included. Patients who had no visit data recorded or had missing start dates for tofacitinib/bDMARD treatment were also not included.

### Statistical and analytical assessment

The primary exposure of interest was an initial prescription for tofacitinib or a bDMARD identified during the sample selection window. The date of the first prescription after the start of the sample selection window served as the study index date and the beginning of the post-index period. Patients who had received a prescription of tofacitinib during the sample selection window were considered part of the ‘tofacitinib group’. All other patients were considered to be in the bDMARD group. Patients who discontinued treatment were followed up for a minimum period of 1 year from their date of index.

Treatment persistence was defined as the time (in consecutive months) from treatment initiation until treatment discontinuation. Duration of treatment of the index b/tsDMARD was estimated using Kaplan–Meier methods. An exploratory and descriptive analysis of the most common reasons for discontinuation was performed.

Treatment pattern evaluation included the percentage of patients receiving monotherapy or conventional synthetic DMARD (csDMARD) combination therapy (methotrexate, hydroxychloroquine, sulfasalazine, leflunomide) at index and at 3-monthly intervals*.* The effectiveness of treatments was assessed using change from baseline (index date) to 18 months in Disease Activity Score-28 three indices including C-reactive protein (DAS28(3)-CRP). This outcome measure was chosen as there was more complete data collected in the medical record to calculate the simpler DAS28(3)-CRP compared to lower levels of data (including patient global assessment) required for calculation of DAS28(4)-CRP, or more disease-specific measures for psoriatic arthritis, such as Disease Activity in Psoriatic Arthritis (DAPSA) or Minimal Disease Activity (MDA). Patients with scores less than 2.6 were classified as being in remission, scores 2.6 to < 3.2 were classified as low disease activity, scores 3.2 to < 5.1 were classified as moderate disease activity and scores ≥ 5.1 were classified as high disease activity [14].

In this retrospective, non-interventional setting, the length of follow-up was variable and also some patients had missing values for some measures at some timepoints. Therefore, all summaries include the number of observations. As this is a descriptive study, no imputation of missing data was performed. In this observational, non-randomised setting, propensity score matching was performed to enhance the comparability of treatment groups however comparisons between groups should still be interpreted with caution (nominal *p*-values are provided to aid interpretation).

### Propensity score matching

Matched analysis sets were constructed to address the observational nature of the data. Propensity score matching was planned between the tofacitinib and each bDMARD group. Propensity score matching increases the comparability of the observed baseline characteristics in patients treated with tofacitinib and bDMARDs. The propensity score is the conditional probability of receiving treatment (e.g. tofacitinib versus IL-17Ai or tofacitinib vs TNFi), which was estimated using logistic regression. Covariates included age group, sex, treatment combination at index and line of therapy (first vs second vs third or greater). Treatment combinations at index and age category were included using indicator variables. The baseline treatment combination covariates were methotrexate monotherapy, methotrexate in combination with other csDMARD(s), csDMARD(s) other than methotrexate and neither methotrexate nor other csDMARD(s) (bDMARD monotherapy).

A calliper width of 0.20 resulted in most tofacitinib patients (> 70%) having a match selected for both matched populations and hence this calliper width was not varied. The success of the matching was determined by examining the propensity score distribution (density plot) in both the original sample and the matched sample, and by comparing standardised difference (in means and proportions) between the matched groups. A difference above 10% (0.1) is generally considered indicative of substantial difference/bias in that covariate.

### Study size

The study size was pragmatic, sampling all available data in the OPAL dataset.

## Results

### Participants

Of 219,812 patients in the OPAL dataset, 16,692 had a diagnosis of PsA and 1486 had been initiated with a b/tsDMARD during the sampling window. Of these, 406 received treatment with tofacitinib, 416 an IL-17Ai (286 with secukinumab and 130 with ixekizumab) and 664 a TNFi (Table 1). There were no missing data on age, combination status or line of therapy (Table 1). Patients had a minimum of 12-month follow-up with a median follow-up of 22 months in each group. The mean (SD) age of tofacitinib, IL-17Ai and TNFi-treated patients were 55.6 (12.6), 52.7 (12.7) and 50.3 (14.6) years, respectively. A slightly higher proportion of female patients was treated with tofacitinib (75.4%) compared with IL-17Ai (61.1%) and TNFi (64.8%). Overall, 19.2% of patients receiving tofacitinib were first line compared with 41.8% of IL-17Ai and 62.8% of TNFi-treated patients. The mean (SD) time from symptom onset to treatment initiation was longer for patients receiving tofacitinib (141.0 (107.9) months) and IL-17Ai (141.6 (106.9) months) compared to TNFi-treated patients (107.3 (97.2) months). At index, patients treated with IL-17Ai had higher physician and patient global assessment of skin visual analogue scale scores than those recorded for tofacitinib or TNFi (Table 1).

**Table 1:** Patient demographics and disease characteristics in the overall population

*N* (%)	Tofacitinib	IL-17Ai	TNFi	All
	406	416	664	1486 (100)
Age at index (years) Mean (SD)	55.56 (12.68) (*n* = 406)	52.65 (12.72) (*n* = 416)	50.32 (14.57) (*n* = 664)	52.40 (13.73) (*n* = 1486)
Median (range)	56.00 (19.00, 93.00) (*n* = 406)	53.00 (20.00, 79.00) (*n* = 416)	51.00 (20.00, 83.00) (*n* = 664)	53.00 (19.00, 93.00) (*n* = 1486)
Gender				
Female	306 (75.4%)	254 (61.1%)	430 (64.8%)	990 (66.6%)
Male	96 (23.6%)	156 (37.5%)	222 (33.4%)	474 (31.9%)
Unassigned	4 (1.0%)	0 (0.0%)	6 (0.9%)	10 (0.7%)
Missing	0 (0.0%)	6 (1.4%)	6 (0.9%)	12 (0.8%)
Time from symptom onset (months) Mean (SD)	140.98 (107.90) (*n* = 182)	141.61 (106.88) (*n* = 172)	107.26 (97.16) (*n* = 267)	126.66 (104.33) (*n* = 621)
Median (range)	110.44 (4.96, 614.60) (*n* = 182)	113.31 (2.14, 481.03) (*n* = 172)	72.22 (0.66, 592.74) (*n* = 267)	94.44 (0.66, 614.60) (*n* = 621)
Disease status				
DAS28(3)-CRP	3.31 (1.39) (*n* = 171)	3.50 (1.46) (*n* = 174)	3.86 (1.47) (*n* = 293)	3.62 (1.46) (*n* = 638)
Total joint count—28, mean (SD)	4.85 (6.24) (*n* = 177)	5.60 (6.66) (*n* = 183)	7.42 (7.78) (*n* = 301)	6.23 (7.17) (*n* = 661)
Total joint count—68, mean (SD)	7.94 (11.23) (*n* = 177)	9.91 (12.00) (*n* = 183)	13.38 (14.62) (*n* = 301)	10.96 (13.26) (*n* = 661)
Swollen joint count—28, mean (SD)	4.22 (5.73) (*n* = 177)	5.25 (6.45) (*n* = 183)	6.82 (7.57) (*n* = 301)	5.69 (6.89) (*n* = 661)
Swollen joint count—66, mean (SD)	6.15 (8.87) (*n* = 177)	8.94 (11.50) (*n* = 183)	11.47 (13.73) (*n* = 301)	9.34 (12.16) (*n* = 661)
Physician skin assessment, mean (SD)	12.36 (19.50) (*n* = 55)	18.09 (20.37) (*n* = 65)	16.32 (21.58) (*n* = 139)	15.92 (20.87) (*n* = 259)
Patient skin assessment, mean (SD)	13.85 (20.95) (*n* = 54)	23.72 (26.22) (*n* = 58)	20.13 (24.82) (*n* = 128)	19.59 (24.50) (*n* = 240)
Previous b/tsDMARDs, *n* (%)				
0	78 (19.2%)	174 (41.8%)	417 (62.8%)	669 (45.0%)
1	101 (24.9%)	119 (28.6%)	145 (21.8%)	365 (24.6%)
2	91 (22.4%)	68 (16.3%)	64 (9.6%)	223 (15.0%)
3	71 (17.5%)	33 (7.9%)	23 (3.5%)	127 (8.5%)
≥ 4	65 (16.0%)	22 (5.3%)	15 (2.3%)	102 (6.9%)
Baseline treatment combinations, *n* (%)				
With methotrexate and other csDMARD*	113 (27.8%)	124 (29.8%)	237 (35.7%)	474 (31.9%)
With methotrexate only	97 (23.9%)	95 (22.8%)	147 (22.1%)	339 (22.8%)
With other csDMARD	57 (14.0%)	60 (14.4%)	105 (15.8%)	222 (14.9%)
b/tsDMARD monotherapy	139 (34.2%)	137 (32.9%)	175 (26.4%)	451 (0.3%)

^*^Other csDMARD includes the following: hydroxychloroquine, sulfasalazine, leflunomide

In the tofacitinib/IL-17Ai propensity score-matched population, there were 269 patients treated with tofacitinib and 269 treated with IL-17Ai. In the tofacitinib/TNFi propensity score-matched population, there were 256 treated with tofacitinib and 256 treated with TNFi. Patient demographics for the overall population are presented in Table 1. Propensity score matching was generally successful, although there were some characteristics that were associated with an inability to find good matches (see Table 2 and 3). Unmatched patients in the tofacitinib/IL-17Ai set were female, tended to be older and were less likely to be on b/tsDMARD monotherapy. Most unmatched patients were on their third or later line of therapy. For the tofacitinib/TNFi set, unmatched patients were also female and older.

**Table 2:** Propensity score matching of tofacitinib and IL-17Ai groups

Factor	Tofacitinib, before matching	IL-17Ai, before matching	Tofacitinib, after matching	IL-17Ai, after matching
*N*	406	416	269	269
Age at index (years), mean (SD)	55.6 (12.7) (*n* = 406)	52.6 (12.7) (*n* = 416)	53.3 (13.3) (*n* = 269)	54.6 (12.7) (*n* = 269)
Age category at index (years)				
18–34 years	26 (6.4%)	43 (10.3%)	25 (9.3%)	17 (6.3%)
35–44 years	50 (12.3%)	69 (16.6%)	50 (18.6%)	45 (16.7%)
45–54 years	109 (26.8%)	120 (28.8%)	66 (24.5%)	68 (25.3%)
55–64 years	114 (28.1%)	111 (26.7%)	67 (24.9%)	79 (29.4%)
65–74 years	82 (20.2%)	58 (13.9%)	49 (18.2%)	47 (17.5%)
75–94 years	25 (6.2%)	15 (3.6%)	12 (4.5%)	13 (4.8%)
Sex				
Male	96 (23.9%)	156 (38.0%)	96 (35.7%)	82 (30.5%)
Female	306 (76.1%)	254 (62.0%)	173 (64.3%)	187 (69.5%)
Combination information				
With Meth + other csDMARD*	113 (27.8%)	124 (29.8%)	70 (26.0%)	68 (25.3%)
With methotrexate only	97 (23.9%)	95 (22.8%)	63 (23.4%)	72 (26.8%)
With other csDMARD	57 (14.0%)	60 (14.4%)	41 (15.2%)	38 (14.1%)
b/tsDMARD monotherapy	139 (34.2%)	137 (32.9%)	95 (35.3%)	91 (33.8%)
Line				
First	78 (19.2%)	174 (41.8%)	77 (28.6%)	67 (24.9%)
Second	101 (24.9%)	119 (28.6%)	99 (36.8%)	79 (29.4%)
≥ Third	227 (55.9%)	123 (29.6%)	93 (34.6%)	123 (45.7%)
DAS28CRP(3), mean (SD)	3.3 (1.4) (*n* = 171)	3.5 (1.5) (*n* = 174)	3.5 (1.4) (*n* = 112)	3.3 (1.4) (*n* = 119)
DAS28CRP(3), median (range)	3.2 (1.3, 7.6) (*n* = 171)	3.2 (1.1, 7.6) (*n* = 174)	3.4 (1.3, 7.6) (*n* = 112)	3.0 (1.1, 7.1) (*n* = 119)
Time since first seen(months), mean (SD)	66.5 (61.5) (*n* = 139)	70.8 (72.1) (*n* = 141)	62.1 (69.6) (*n* = 92)	(74.2) (*n* = 102)

^*^Other csDMARD includes the following: hydroxychloroquine, sulfasalazine, leflunomide

**Table 3:** Propensity score matching of tofacitinib and TNFi groups

Factor	Tofacitinib, before matching	TNFi, before matching	Tofacitinib, after matching	TNFi, after matching
*N*	406	664	256	256
Age at index (years), mean (SD)	55.6 (12.7) (*n* = 406)	50.3 (14.6) (*n* = 664)	53.0 (13.5) (*n* = 256)	53.9 (13.7) (*n* = 256)
Age category at index (years)				
18–34 years	26 (6.4%)	116 (17.5%)	25 (9.8%)	23 (9.0%)
35–44 years	50 (12.3%)	115 (17.3%)	50 (19.5%)	40 (15.6%)
45–54 years	109 (26.8%)	155 (23.3%)	64 (25.0%)	65 (25.4%)
55–64 years	114 (28.1%)	160 (24.1%)	61 (23.8%)	67 (26.2%)
65–74 years	82 (20.2%)	90 (13.6%)	42 (16.4%)	45 (17.6%)
75–94 years	25 (6.2%)	28 (4.2%)	14 (5.5%)	16 (6.2%)
Sex				
Male	96 (23.9%)	222 (34.0%)	77 (30.1%)	64 (25.0%)
Female	306 (76.1%)	430 (66.0%)	179 (69.9%)	192 (75.0%)
Combination information				
With Meth + other csDMARD*	113 (27.8%)	237 (35.7%)	76 (29.7%)	70 (27.3%)
With methotrexate only	97 (23.9%)	147 (22.1%)	59 (23.0%)	68 (26.6%)
With other csDMARD	57 (14.0%)	105 (15.8%)	50 (19.5%)	47 (18.4%)
b/tsDMARD monotherapy	139 (34.2%)	175 (26.4%)	71 (27.7%)	71 (27.7%)
Line				
First	78 (19.2%)	417 (62.8%)	77 (30.1%)	79 (30.9%)
Second	101 (24.9%)	145 (21.8%)	101 (39.5%)	82 (32.0%)
≥ Third	227 (55.9%)	102 (15.4%)	78 (30.5%)	95 (37.1%)
DAS28CRP(3), mean (SD)	3.3 (1.4) (*n* = 171)	3.9 (1.5) (*n* = 293)	3.5 (1.4) (*n* = 106)	3.5 (1.4) (*n* = 118)
DAS28CRP(3), median (range)	3.2 (1.3, 7.6) (*n* = 171)	3.8 (1.3, 7.1) (*n* = 293)	3.4 (1.3, 6.6) (*n* = 106)	3.3 (1.4, 7.1) (*n* = 118)
Time since first seen(months), mean (SD)	66.5 (61.5) (*n* = 139)	46.4 (60.4) (*n* = 202)	53.0 (58.8) (*n* = 86)	54.2 (60.5) (*n* = 86)

^*^Other csDMARD includes the following: hydroxychloroquine, sulfasalazine, leflunomide

### Treatment patterns

At treatment initiation, approximately 25% of TNFi-treated patients and one-third of patients treated with tofacitinib and IL-17Ai were prescribed b/tsDMARD monotherapy. Another third received treatment in combination with both methotrexate and another csDMARD for TNFi and ~ 30% for tofacitinib and IL-17i (Table 4). By 12 months, approximately 1 in 5 patients had ceased treatment with their b/tsDMARD, almost one-quarter were receiving b/tsDMARD monotherapy, just under 20% were receiving methotrexate with a csDMARD, and 20% were receiving methotrexate alone (Table 4). See also Supplementary Fig. 1.

**Table 4:** Treatment patterns at initiation and after 12 months in overall population

Timepoint	Combination	Tofacitinib	IL-17Ai	TNFi
*N*		406	416	664
0 to < 12 weeks	With methotrexate + other csDMARD	113 (27.8%)	124 (29.8%)	233 (35.1%)
	With methotrexate only	98 (24.1%)	96 (23.1%)	150 (22.6%)
	With other csDMARD	58 (14.3%)	60 (14.4%)	107 (16.1%)
	b/tsDMARD monotherapy	137 (33.7%)	136 (32.7%)	173 (26.1%)
	No further follow-up*	0 (0.0%)	0 (0.0%)	1 (0.2%)
12 to < 24 weeks	With methotrexate + other csDMARD	92 (22.7%)	99 (23.8%)	196 (29.5%)
	With methotrexate only	92 (22.7%)	98 (23.6%)	154 (23.2%)
	With other csDMARD	47 (11.6%)	50 (12.0%)	92 (13.9%)
	b/tsDMARD monotherapy	107 (26.4%)	120 (28.8%)	148 (22.3%)
	Stopped	28 (6.9%)	8 (1.9%)	14 (2.1%)
	No further follow-up*	40 (9.9%)	41 (9.9%)	60 (9.0%)
24 to < 52 weeks	With methotrexate + other csDMARD	69 (17.0%)	66 (15.9%)	121 (18.2%)
	With methotrexate only	77 (19.0%)	81 (19.5%)	145 (21.8%)
	With other csDMARD	32 (7.9%)	35 (8.4%)	64 (9.6%)
	b/tsDMARD monotherapy	97 (23.9%)	105 (25.2%)	142 (21.4%)
	Stopped	89 (21.9%)	84 (20.2%)	128 (19.3%)
	No further follow-up*	42 (10.3%)	45 (10.8%)	64 (9.6%)
52 to < 76 weeks	With methotrexate + other csDMARD	43 (10.6%)	41 (9.9%)	70 (10.5%)
	With methotrexate only	54 (13.3%)	58 (13.9%)	102 (15.4%)
	With other csDMARD	26 (6.4%)	22 (5.3%)	44 (6.6%)
	b/tsDMARD monotherapy	48 (11.8%)	78 (18.8%)	94 (14.2%)
	Stopped	156 (38.4%)	128 (30.8%)	232 (34.9%)
	No further follow-up*	79 (19.5%)	89 (21.4%)	122 (18.4%)
76 to < 104 weeks	With methotrexate + other csDMARD	24 (5.9%)	16 (3.8%)	32 (4.8%)
	With methotrexate only	30 (7.4%)	32 (7.7%)	59 (8.9%)
	With other csDMARD	14 (3.4%)	14 (3.4%)	26 (3.9%)
	b/tsDMARD monotherapy	32 (7.9%)	39 (9.4%)	50 (7.5%)
	Stopped	177 (43.6%)	167 (40.1%)	282 (42.5%)
	No further follow-up*	129 (31.8%)	148 (35.6%)	215 (32.4%)

Note: Only includes patients who were on their index treatment in that period and with concomitant medical information available. Patients with no stop date for index treatment are included only up to their last visit

^*^No further follow-up refers to patients who have no further visit information available

### Treatment persistence

**Fig. 1:**
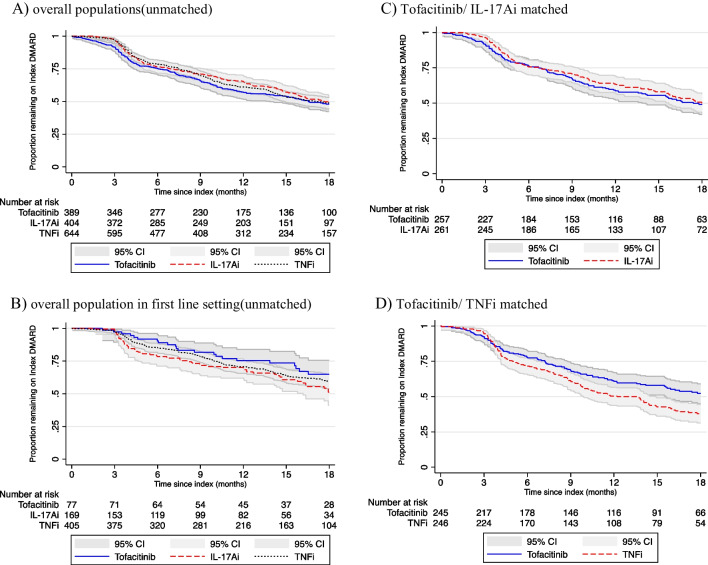
Treatment persistence in **A** overall population (unmatched), **B** overall population in first-line setting (unmatched), **C** tofacitinib/IL-17Ai matched and **D** tofacitinib/TNFi matched

Treatment persistence was similar across all treatment groups (Fig. 1) in the unmatched population with a median persistence of 16.5 months (95% CI 13.8 to 19.5 months) in the tofacitinib group; 17.7 months (95% CI 15.8 to 19.6 months) in the IL-17Ai group and 17.2 months (95% CI 14.9 to 20.5 months) in the TNFi group (Fig. 1A). As might be expected, persistence in the first-line setting was longer: 22.0 months (95% CI 18.7 to not reached) in the tofacitinib group, 18.2 months (95% 16.2 to 22.6 months) in the IL-17Ai group and 26.1 months (95% CI 20.8 to not reached) in the TNFi group (Fig. 1B). In the tofacitinib/IL-17Ai matched population, median persistence was similar, 17.4 months (95% CI 13.8 to 20.3 months) in the tofacitinib group and 18.0 (95% CI 15.8 to 19.6 months) in the IL-17Ai group (Fig. 1C). In the tofacitinib/TNFi matched population, persistence was longer in the tofacitinib group (18.7 months, 95% CI 15.6 to 21.4 months) compared with the TNFi group (12.2 months, 95% CI 19.9 to 14.9 months; Fig. 1D).

Around half of tofacitinib (*n* = 139, 50.5%), IL-17Ai (*n* = 157 (53.4%) and TNFi (*n* = 241, 50.3%) patients ceased their index b/tsDMARD. The reason for discontinuing treatment was documented by the rheumatologist in the patient’s EMR at the time of the decision from a pre-defined menu. Lack of efficacy (including primary failure, secondary failure and partial response) was recorded in 28% of patients treated with tofacitinib, 28% of patients treated with an IL-17Ai and 31% of patients treated with TNFi. Adverse reaction was recorded in 15.1%, 7.0% and 6.2% of patients treated with tofacitinib, IL-17Ai and TNFi, respectively and ‘better alternative’ was given as a reason for ceasing in 11.5% of tofacitinib patients, 8.9% of IL-17Ai and 10.4% of TNFi-treated patients. (A full list of reasons for discontinuation is provided in supplementary Table 1.)

### Treatment effectiveness

**Fig. 2:**
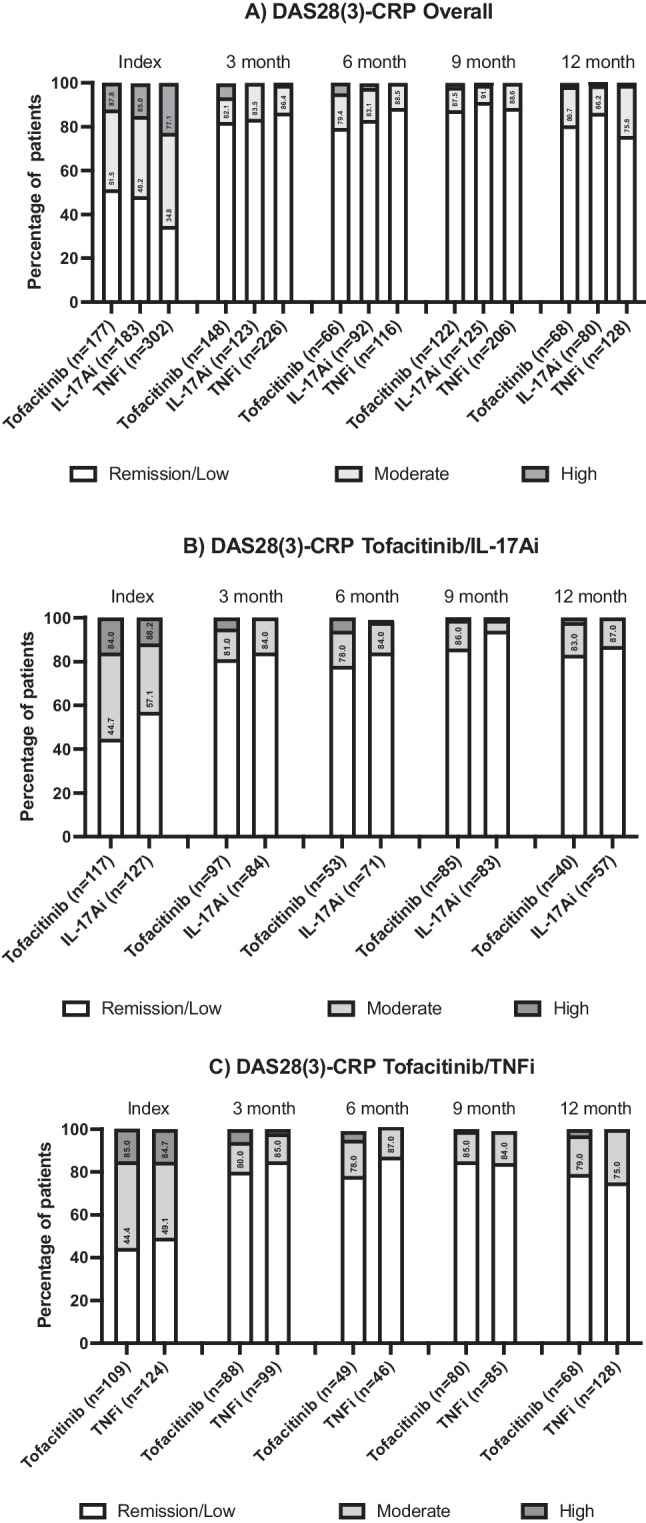
Percentage of patients achieving DAS28(3)-CRP disease activity levels for **A** overall population (all eligible patients three groups, unmatched), **B** tofacitinib/IL-17Ai matched and **C** tofacitinib/TNFi matched (data at index, 3, 6, 9, 12 months)

Treatment effectiveness was assessed using the DAS28(3)-CRP measure. This is a three-variable measure based on swollen and tender joint counts and CRP but lacks the patient global assessment that is used in the DAS28-CRP. At index, 51.5%, 48.2% and 34.8% of patients treated with tofacitinib, IL17Ai and TNFi (respectively) were in remission or LDA based on their DAS28(3)-CRP scores, which increased to approximately 80% of patients by 3 months and was maintained to 12 months (Fig. 2A). There appear to be some numerical differences in efficacy at 6 months between both tofacitinib and IL-17Ai, with 78% of tofacitinib patients and 84% of IL-17Ai patients in remission or LDA (Fig. 2B) and 78% of tofacitinib and 87% of TNFi patients (Fig. 2C) achieving remission or LDA at 6 months. However, this difference did not persist and may be related to there being less numbers of patients with data at the 6-month timepoint.

## Discussion

Our study reports the treatment persistence and treatment patterns of a large cohort of patients with PsA treated with b/tsDMARDs in a real-world setting in Australia. Almost 1500 patients are included from the overall dataset. Patients receiving tofacitinib were predominantly in later lines of treatment compared to those on IL-17Ai and TNFi. Consequently, their median age was slightly higher, at 55 years compared to 52 and 50 years for IL-17Ai and TNFi, respectively. The utilisation of newer treatments in later lines of therapy is a common trend. Older and more established bDMARDs like TNFi are frequently employed as the first-line therapy in PsA, likely due to the accrued level of familiarity rheumatologists have with their efficacy, safety profile, as well as their potential short- and long-term adverse events. Furthermore, patients experiencing multiple treatment failures may have additional factors at play, such as the presence of conditions like fibromyalgia, a high body mass index, and comorbidities, which can complicate their management. However, we should note that our study did not delve into factors associated with challenging-to-treat conditions.

Treatment persistence was found to be similar across the different treatment types in an unmatched PsA population, with median persistence of approximately 17 months in all lines of therapy, with longer persistence found in the first-line setting. This is similar to that observed in Swedish [15], French [16] and Israeli [17] cohorts of patients with PsA. This contrasts with rheumatoid arthritis patients from the OPAL dataset in whom median treatment persistence was approximately double that of our PsA cohort [18]. Differences in persistence between disease states have been reported previously, although the reasons for observed differences are unknown [19]. In the analysis of the matched population receiving tofacitinib and IL-17Ai therapies, the median duration of persistence was found to be comparable between the two groups while in the matched population of tofacitinib and TNFi recipients, the tofacitinib group was found to have longer median persistence. Limited data exists in the literature comparing the persistence of matched tofacitinib and TNFi treatment groups, so validation of these results from other sources will be of interest.

In Australia, tofacitinib, IL-17Ai and TNFi are indicated for treatment of PsA alone or in combination with csDMARDs for patients who have had an inadequate response to two prior DMARD therapies. Around one-third of patients treated with a non-TNFi (tofacitinib or IL-17Ai) were treated as monotherapy, while slightly less, ~ 25% of TNFi-treated patients, were treated as monotherapy. Recently, the role of methotrexate in severe PsA has been questioned due to the scarcity of supporting data which has mainly come from small inconclusive trials [20, 21]. The SEAM-PsA study in 2019 demonstrated that etanercept exhibited superior efficacy to MTX and the addition of methotrexate to etanercept made no substantial impact on the overall effectiveness of etanercept monotherapy [22]. The European Alliance of Associations for Rheumatology (EULAR) recommendations for the pharmacological treatment of PsA however continue to place MTX and other csDMARDs at the top of the treatment algorithm [23]. Although limited data comparing tofacitinib monotherapy vs combination therapy exist, a recent real-world study from the United States of America reported that persistence rates for monotherapy and combination tofacitinib therapy in PsA were similar after 6 months of treatment [24]. Furthermore, side effects from methotrexate such as fatigue commonly lead to discontinuation [25] and thus in practice treatment with b/tsDMARD monotherapy is common, as was observed in our study.

From the data that were available, using DAS28-3(CRP) as a surrogate for disease activity, on average, most patients had moderate to high disease activity at the time of index. All three treatments (tofacitinib, IL17Ai and TNFi) in the overall population and in the tofacitinib/IL-17Ai and tofacitinib/TNFi matched populations effectively increased the proportion of patients in remission or LDA to around 80% by 3 months of treatment, which was maintained out to 12 months.

Although persistence is a multifaceted measure that considers factors beyond treatment efficacy, including safety, tolerability and patient satisfaction or preferences, it is also often used as a surrogate for efficacy in the real world [26–28]. Using persistence as a broad marker of efficacy here, there were no differences between the 3 treatment groups. This is consistent with the findings of a network meta-analysis which found similar clinical efficacy for tofacitinib when compared with bDMARDs [29]. In this study, we used propensity scoring to match treated populations and found that the persistence on tofacitinib was greater than that on TNFi but not IL-17Ai. We are not aware of other studies that have directly compared persistence on tofacitinib with bDMARDs using this approach.

### Limitations

There are several limitations to our study. Given the nature of data collection in this study, missing data are inevitable, and thus some assessments were hampered by missing data. This led to the use of a non-disease specific measure, the DAS28(3)-CRP, being used as an outcome measure for disease activity. While the DAS28 is commonly used to measure disease activity in PsA patients, it was not originally designed for this purpose. This study was unable to use a more PsA-specific measure, such as the DAPSA, due to limitations in real-world clinical practice. In Australia, the routine collection of DAPSA scores and skin assessments is not widespread, and only a subset of rheumatologists incorporates this measure in their regular clinical evaluation of patients. These findings underscore the challenges associated with using more specialised measures in routine clinical care and highlight some of the limitations of real-world studies.

The direction of missing data bias depends on the measure being assessed. It might be reasonable to expect, for example, that those with troublesome skin manifestations are more likely to have a physician or patient global assessment of skin performed. The sample size, variables and study duration were selected to minimise the impact of this. As suggested previously [18], we assume, however, given the source of this data, that data are likely to be missing at random, as important clinical information is likely to have been reported within the patient’s medical record. The results from the matched analyses may not be generalisable to the patient population as there were a substantial proportion (around one-third) of tofacitinib patients for whom good matches on other treatments could not be found. In addition, not all variables that could potentially influence choice of prescription were collected and hence could not be included in the propensity score model (e.g. dactylitis, enthesitis, severe skin involvement, axial involvement, involvement of lung, cardiovascular, skin). This may lead to biased estimates of differences between groups.

## Conclusions

In this analysis of a large real-world dataset in Australia, tofacitinib was seen to be more commonly utilised in later lines of therapy and in a slightly higher proportion of female patients than IL-17Ai or TNFi. Treatment persistence was similar for tofacitinib, IL-17Ai and TNFi in the overall population, with longer persistence than TNFi in a matched population.

### Supplementary Information

Below is the link to the electronic supplementary material.

Supplementary file1 (DOCX 53 KB)

## Data Availability

Data collection is based on opt-out patient consent, and patients have consented to their data being made available to OPAL Rheumatology only. Requests for access to summary statistics will be considered by the OPAL Scientific Review Committee.
