# Supplementary material for: Real-world evaluation of persistence, effectiveness and usage patterns of tofacitinib in treatment of psoriatic arthritis in Australia
Source: Clin Rheumatol. 2024 Mar 9;43(5):1579–89. doi: 10.1007/s10067-024-06930-7 (PMC11018696; doi:10.1007/s10067-024-06930-7)
Supplement: Supplementary file 1 — Supplementary file1 (DOCX 53 KB) [file 10067_2024_6930_MOESM1_ESM.docx]

# Supplemental Text


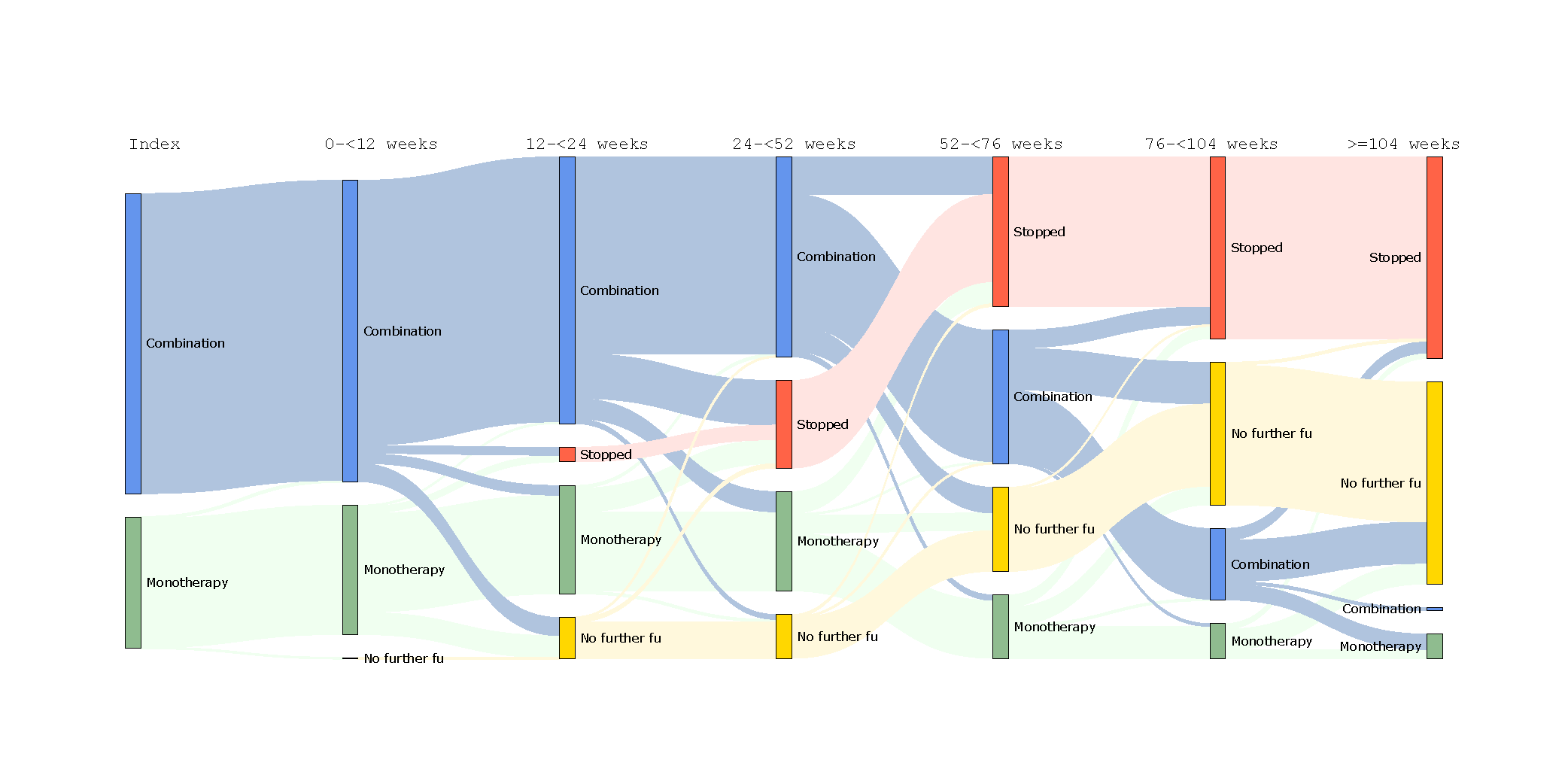


Supplementary Figure 1: Flow between monotherapy and combination therapy (all eligible patients across treatment groups; n=1486)

Fu=follow-up

| Supplementary Table S1. Reasons for discontinuation | | | |
| --- | --- | --- | --- |
| Factor | Tofacitinib | IL-17Ai | TNFi |
| N | 275 | 294 | 479 |
| Ceased index bDMARD treatment | 139 (50.5%) | 157 (53.4%) | 241 (50.3%) |
| Cessation reason |  |  |  |
| Adverse Reaction | 21 (15.1%) | 11 (7.0%) | 15 (6.2%) |
| Lack of Efficacy (including primary failure, secondary failure, and partial response) | 39 (28%) | 44 (28.0%) | 74 (30.7%) |
| Better Alternative | 16 (11.5%) | 14 (8.9%) | 26 (10.8%) |
| Completed treatment / no longer required* | 36 (25.9%) | 69 (43.9%) | 85 (35.3%) |
| Completed Treatment – De-escalation/Drug free remission | 0 (0.0%) | 1 (0.6%) | 1 (0.4%) |
| Other/Uncertain | 5 (3.6%) | 7 (4.5%) | 4 (1.7%) |
| Patient non-adherence | 2 (1.4%) | 3 (1.9%) | 2 (0.8%) |
| Contra Indication | 3 (2.2%) | 0 (0.0%) | 0 (0.0%) |
| Missing - No reason recorded | 17 (12.2%) | 8 (5.1%) | 34 (14.1%) |
| Percentages for cessation reasons calculated using denominator of patients who discontinued only.  *’Completed treatment/no longer required’ does not mean drug-free remission in this context, Often selected when no other suitable option available in the dropdown list. | | | |
